# The Relationship between Non-Suicidal Self-Injury and the UPPS-P Impulsivity Facets in Eating Disorders and Healthy Controls

**DOI:** 10.1371/journal.pone.0126083

**Published:** 2015-05-20

**Authors:** Laurence Claes, Mohammed A. Islam, Ana B. Fagundo, Susana Jimenez-Murcia, Roser Granero, Zaida Agüera, Elisa Rossi, José M. Menchón, Fernando Fernández-Aranda

**Affiliations:** 1 CIBER Fisiología de la Obesidad y Nutrición (CIBERobn), Instituto Salud Carlos III, Barcelona, Spain; 2 Faculty of Psychology and Educational Sciences, University of Leuven, Leuven, Belgium; 3 Department of Psychiatry, University Hospital of Bellvitge-IDIBELL, Barcelona, Spain; 4 Department of Clinical Sciences, School of Medicine, University of Barcelona, Barcelona, Spain; 5 Departament de Psicobiologia i Metodologia, Universitat Autònoma de Barcelona, Barcelona, Spain; 6 CIBER Salud Mental (CIBERSAM), Instituto Salud Carlos III, Barcelona, Spain; Central Institute of Mental Health, GERMANY

## Abstract

In the present study, we investigated the association between Non-Suicidal Self-Injury (NSSI) and the UPPS-P impulsivity facets in eating disorder patients and healthy controls. The prevalence of NSSI in eating disorder (ED) patients ranged from 17% in restrictive anorexia nervosa (AN-R) patients to 43% in patients with bulimia nervosa (BN). In healthy controls (HC), the prevalence of NSSI was 19%. Eating disorder patients from the binge eating/purging type showed significantly more NSSI compared to restrictive ED and HC participants. Binge-eating/purging ED patients also scored significantly higher on Negative/Positive Urgency, Lack of Premeditation and Lack of Perseverance compared to HC and restrictive ED patients. Comparable findings were found between ED patients and HC with and without NSSI; ED patients and HC with NSSI scored significantly higher in four of the five UPPS-P dimensions compared to participants without NSSI; Sensation Seeking was the exception. Finally, the presence of NSSI in HC/ED patients was particularly predicted by low levels of Perseverance. Therefore, the treatment of ED patients with NSSI certainly needs to focus on the training of effortful control.

## Introduction

Non-suicidal self-injury (NSSI) refers to the deliberate and direct injury of one’s own body tissue without suicidal intent [1], such as scratching, cutting, hitting and burning oneself. The prevalence of NSSI in eating disorder patients is high ranging between 25.4% and 55.2% [2,3]. The prevalence of NSSI generally appears to be higher in patients with bulimia nervosa (BN) and anorexia nervosa binge eating/purging subtype (AN-BP) compared to patients with anorexia nervosa restrictive subtype (AN-R) [3]. In community samples, the prevalence of NSSI is estimated to be around 18% in adolescents and young adults [4]. According to Peterson and Fischer [5], the link between NSSI and BN in community and clinical samples, can be the desire to quickly reduce negative affect. Both NSSI and BN have been conceptualized as the result of deficits in impulse control, particularly in response to distress [5]. For example, the escape model of Heatherton and Baumeister [6], which is applied to both BN symptoms and NSSI [7], proposed that individuals engage in BN symptoms and NSSI in order to make themselves less aware of negative affect. Both BN and NSSI dull painful feelings and are thus negatively reinforcing [8]. Recent developments in the study of impulsivity theoretically clarify the role of reactivity to negative affect in impulsive action [5]. One of these models, the UPPS model of Whiteside and Lynam [9], originally described four distinct personality pathways to impulsive behavior: Negative Urgency, Lack of Perseverance, Lack of Premeditation and Sensation Seeking. Negative Urgency refers to the tendency to act impulsively when experiencing negative affect. Lack of Perseverance assesses a tendency to give up due to fatigue, boredom and frustration. Lack of Premeditation refers to the tendency to act without consideration of the potential consequences of the behavior. And finally, Sensation Seeking measures the person’s tendency to pursue actions that are perceived as exciting and novel [9]. In 2007, Cyders et al. [10] have identified an important additional dimension, Positive Urgency, which refers to the tendency to act impulsively when experiencing positive affect. To assess these five pathways of impulsive behavior Whiteside and Lynam [9] developed the UPPS-P Impulsive Behavior Scale.

In recent years, several studies have investigated the association between eating disorder symptoms and the UPPS(-P) dimensions in community and clinical samples. In a review paper of Fischer et al. [11,12], Negative Urgency was positively associated with BN symptoms with moderate to high effect sizes, while the relationship of the other impulsivity dimensions and BN showed small effect sizes. Additionally, Anestis et al. [8] replicated the positive association between Negative Urgency and BN symptoms in a clinical sample and they showed that changes in levels of negative urgency over time were able to predict associated changes in BN symptoms [13]. Finally, Claes, Vandereycken and Vertommen [14] reported that BN patients consistently showed more Negative Urgency and Sensation Seeking and less Premeditation and Perseverance than restrictive AN (AN-R) patients, with the binge eating/purging AN (AN-BP) patients being situated in between.

Similarly, several studies have investigated the association between the presence/absence of NSSI and the UPPS-P dimensions in community and clinical samples. Glenn and Klonsky [15], showed that college students with NSSI scored significantly higher on Negative Urgency, Lack of Premeditation and Sensation Seeking compared to students without NSSI with the strongest effect in Negative Urgency. Lack of Perseverance predicted more recent and frequent NSSI. Although the UPPS dimensions showed significant differences between students with NSSI and without NSSI. The UPPS dimensions were not able to prospectively predict future NSSI in this same sample of college students [16]. More recently, Claes and Muehlenkamp [17] related the five UPPS-P dimensions to the presence/absence of NSSI in a high school population, and found significantly positive associations between NSSI and Positive and Negative Urgency (the tendency to act impulsively while experiencing positive and negative affect). Additionally, Bresin, Carter and Gordon [18] studied the interaction between emotional experiences (sadness and guilt) and the Negative Urgency impulsivity-dimension in college students. It was found that for individuals high on Negative Urgency, sadness was a significant predictor of NSSI urge whereas this was not the case with individuals who scored low on Negative Urgency. They showed that a specific negative affective state (e.g., sadness) was stronger related to NSSI urge among individuals high in negative urgency (trait). Finally, Lynam, Miller, Miller, Bornovalova and Lejuez [19] investigated the association between NSSI and the UPPS dimensions in a clinical sample of addicted patients and found that Negative Urgency, Lack of Premeditation and their interaction predicted the presence of NSSI in this sample. The highest probability to engage in NSSI was found in patients who reported high levels of Negative Urgency in combination with low levels of Premeditation.

The aim of the present study was to integrate these two lines of research into one study. First, we wanted to investigate the prevalence of NSSI in different subtypes of ED patients and healthy controls. Based on the literature we expected prevalence rates between 25.4% and 55.2%, with a slightly higher prevalence of binge-eating/purging ED patients [3]. Second, we investigated differences in the five UPPS-S dimensions in subtypes of ED patients and a healthy control group. Based on the literature, we certainly expected higher scores on the Negative/Positive Urgency dimensions in binge-eating/purging patients compared to restrictive ED patients and healthy controls [12,14]. Third, we compared ED patients/healthy controls with and without NSSI on the UPPS-P dimensions and expected that ED patients/healthy controls with NSSI would score higher on Positive/Negative Urgency and Lack of Premeditation compared to those without NSSI [15]. Finally, we investigated which of the five UPPS-P dimensions as well as their interactions were able to predict the presence/absence of NSSI in ED patients/healthy controls. Driven by earlier findings, we hypothesized that Negative Urgency, Lack of Premeditation and their interaction would be the strongest predictors of NSSI in our ED/healthy control samples [19].

## Materials and Method

### Participants

The sample was recruited between January 2012 and July 2014. The total sample consisted of 481 participants: 113 healthy controls (26 males) and 368 (17 males) ED patients. Given the small number of male participants, they were removed from our sample, leaving a total of 438 female participants. Additionally, 43 ED patients with Binge Eating Disorder (BED) were removed, due to the fact that they were significantly older (M = 39.60 years, SD = 11.02) and weighed significantly more (BMI: M = 38.63, SD = 8.22) compared to other ED patients and that the relevant literature did not pertain to BED. Finally, from the remaining 395 participants, 57 (3 HC, 54 ED) were removed due to missing data on the variable Non-Suicidal Self-Injury. The final sample consisted of 338 female participants: 84 HC and 254 ED patients.

The ED patients were consecutive referrals for assessment and treatment at the Department of Psychiatry of the University Hospital of Bellvitge in Barcelona. All participants were diagnosed according to the DSM-IV-TR diagnostic criteria [20] by means of the SCID-I [21], conducted by experienced psychologists and psychiatrists. About 18.5% (n = 47) of the patients with ED were diagnosed with Anorexia Nervosa-Restrictive subtype (AN-R), 10.2% (n = 26) with Anorexia Nervosa Binge-Purge subtype (AN-BP), 46.5% (n = 118) with BN and 24.8% (n = 63) with EDNOS (eating disorder not otherwise specified). The mean duration of ED was 8.29 years (*SD* = 8.38), and statistical differences appeared in an ANOVA comparison [*F*_(3, 242)_ = 3.358, *p* =. 020]: significant pairwise comparisons were obtained for AN-R versus AN-BP patients (*means* 5.47 vs 11.8 years; *p =*. 003) and AN-R vs BN patients (*means* 5.47 vs 8.79; *p =*. 024). Mean age was 28.15 (SD = 9.47) in ED and 21.33 (SD = 6.63) in HC [F _(1,335)_ = 37.00, p<.001]. Between the groups there were no significant differences found in mean BMI [ED: 21.94 kg/m2 (SD 3.13); HC: 21.9 kg/m2 (SD 3.13); *F*_(1, 322)_ = 0.00, *p* =. 982]. Within the ED subtypes, no significant differences for age were found (*F*_(3,252)_ = 0.89, *p* =. 449). However, within ED subtypes as expected, significant results were obtained in the ANOVA for the BMI mean comparison (*F*_(3,241)_ = 36,71, *p* <. 001): pairwise comparisons showed significant contrasts for AN-R compared to BN (*means* 16.6 vs 24.6; *p* <. 001), AN-R compared to EDNOS (*means* 16.6 vs 22.8; *p* <. 001), AN-BP compared to BN (*means* 17.2 vs 24.6; *p* <. 001), AN-BP compared to EDNOS (*means* 17.2 vs 22.8; *p* <. 001) and BN compared to EDNOS (*means* 24.6 vs 22.8; *p* =. 024).

The final sample of female HC consisted of 84 participants. Healthy controls were recruited via advertisements in the Health Sciences Bellvitge Campus of the University of Barcelona. All HC were from the same catchment areas as the ED patients. Prior to questionnaire assessment, HC were interviewed about their lifetime history of health or mental diseases (including EDs) by means of the General Health Questionnaire-28 (GHQ-28) [22] and specific ED criteria. Exclusion criteria for the HC were: (a) having suffered from a lifetime Axis I mental disorder and (2) having an age below 16 and above 60.

All participants were informed about the research procedures and gave informed consent in writing and signed. Procedures were approved by the Ethical Committee of the University Hospital of Bellvitge.

### Instruments

Information concerning socio-demographic variables (e.g., education level, marital status, employment status) and ED characteristics (e.g. weight, length, BMI, age of onset, duration of ED) was assessed by means of a semi-structured clinical interview.

The presence/absence of NSSI was investigated by a 1-item question asking, “Have you ever engaged in self-injury without the intent to die?” Using a single-item measure of NSSI is common in NSSI research and has been shown to render consistent estimates of prevalence [4].

To assess impulsivity facets the Spanish version of the UPPS-P Impulsivity Scale [9,23] was used. The UPPS-P Impulsivity Scale [9] is a 59-item inventory to measure five distinct features of impulsive behavior: Negative Urgency, Lack of Perseverance, Lack of Premeditation, Sensation Seeking and Positive Urgency. Each item of the UPPS-P is rated on a 4-point Likert scale ranging from 1-‘strongly agree’ to 4-‘strongly disagree’. The Cronbach’s alphas [24] of the scales in the present study are as follows: Negative Urgency (α =. 87), Lack of Perseverance (α =. 87), Lack of Premeditation (α =. 79), Sensation Seeking (α =. 89) and Positive Urgency (α =. 93).

### Statistical Analyses

All statistical analyses were conducted using the Statistical Package for the Social Sciences, SPSS version 22.0 for Windows. To explore the association between the presence/absence of NSSI and HC/ED subtypes the χ^2^ test statistic was used. Differences in UPPS-P impulsivity facet scores in function of the presence/absence of NSSI, HC/ED subtypes (HC, AN-R, AN-BP, BN and EDNOS) and their interaction were investigated by means of a multivariate analysis of variance (ANOVA) controlled for the participants’ age. For non-significant interaction NSSI*Group, main effects were estimated and interpreted, while single effects were obtained and interpreted for significant interaction parameters. One independent ANOVA procedure was conducted for each UPPS-P scale and the Simes-Bonferroni method was used to control Type-I error due to multiple comparisons [25]. This corrective procedure is included into the family-wise error rate stepwise procedures, and offers more powerful test than the classical Bonferroni correction.

Finally, a logistic regression model valued the predictive capacity of the UPPS-P impulsivity facet(s) scores and their interactions on the presence of the NSSI behavior, adjusted by the covariates age and diagnostic subtype. One logistic model was obtained, including in a first block-step the covariates age and diagnostic group, in a second block-step the UPPS-P scores and in a third block-step the interactions between the UPPS-P dimensions.

## Results

### Prevalence of NSSI in HC/ED subtypes

Table 1 displays the lifetime prevalence of NSSI in HC and ED subtypes. Overall, a significant association is found between the presence of NSSI and HC/ED subtypes [χ^2^_(4)_ = 20.13, *p*<.001]. Post-hoc comparison between diagnostic subtypes indicated that prevalence of NSSI in the HC group was not significantly different from the AN-R group (OR = 1.15, *p* =. 774), but the NSSI prevalence was lower in HC when compared to AN-BP (OR = 2.65, *p* =. 046), BN (OR = 3.24, *p*<.001.) and EDNOS (OR = 2.80, *p* =. 007). Among the ED subtypes, AN-R showed lower prevalence than AN-BP (OR = 3.05, *p* =. 046), BN (OR = 3.71, *p* =. 002) and EDNOS (OR = 3.21, *p* =. 012), while equal prevalence rates were obtained for AN-BP, BN and EDNOS.

**Table 1:** Prevalence rates for lifetime NSSI and comparison between diagnostic group.

Diagnostic	Sample	Lifetime NSSI prevalence			Significant pairwise comparison	
Group	size *(N)*	Count	%	95% CI	Comparison	OR (95% CI)
HC	84	16	19.0%	12.1% to 28.7%	HC < AN-BP	2.65 (1.02 to 6.94)
AN-R	47	8	17.0%	8.88% to 30.1%	HC < BN	3.24 (1.68 to 6.23)
AN-BP	26	10	38.5%	22.4% to 57.5%	HC < EDNOS	2.80 (1.33 to 5.88)
BN	118	51	43.2%	34.6% to 52.2%	AN-R < AN-BP	3.05 (1.02 to 9.12)
EDNOS	63	25	39.7%	28.5% to 52.0%	AN-R < BN	3.71 (1.60 to 8.63)
		Group: X_(4)_ = 20.13, *p*<.001			AN-R < EDNOS	3.21 (1.29 to 7.99)

CI = Confidence Interval, OR = Odds Ratio, HC = Healthy Controls, AN-R = Anorexia Nervosa Restrictive Subtype, AN-BP = Anorexia Nervosa Binge-eating/Purging type, BN = Bulimia Nervosa, EDNOS = Eating Disorder Not Otherwise Specified

### Comparison of UPPS-P impulsivity facets between HC/ED participants with (out) NSSI

**Fig 1:**
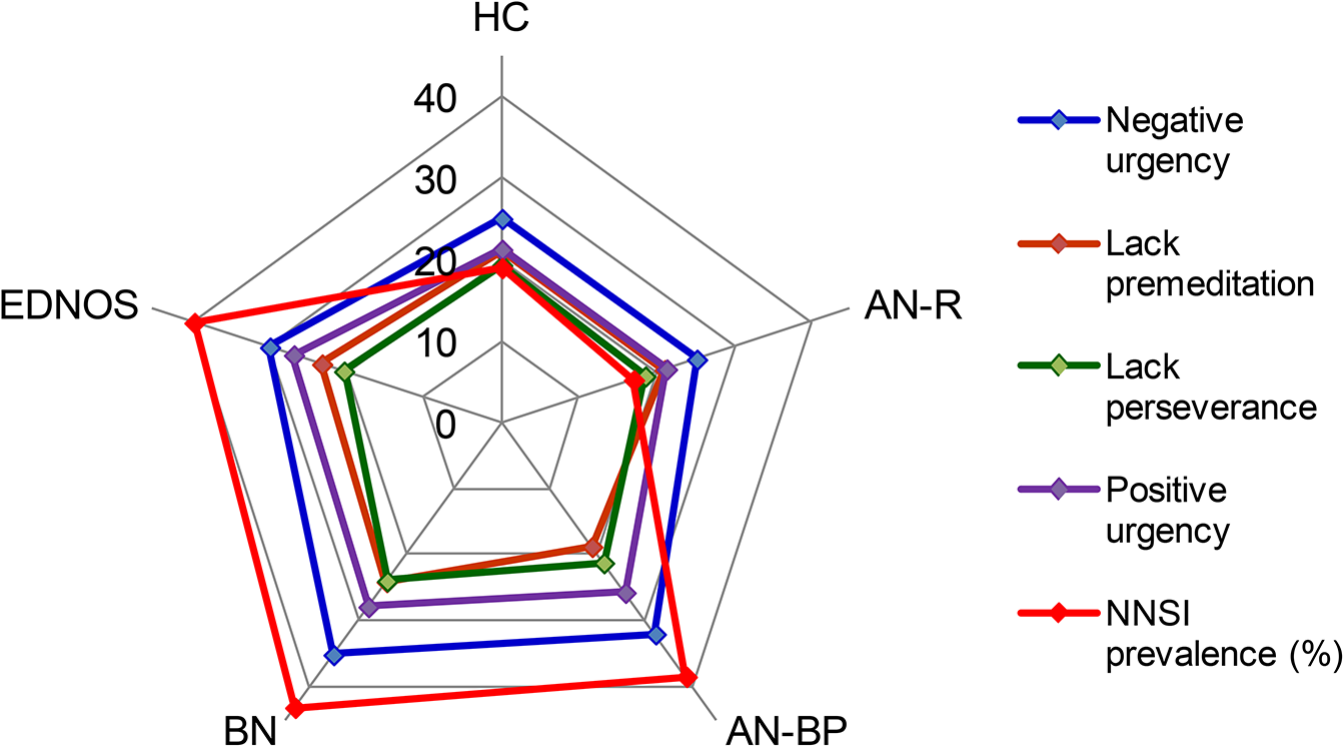
Radar chart for the UPPS-P mean scores and the lifetime prevalence of Non-Suicidal Self-Injury (NSSI), stratified by the diagnostic subtypes [Healthy Controls (HC), Anorexia Nervosa-Restrictive type (AN-R), Anorexia Nervosa-Binge-eating/Purging type (AN-BP), Bulimia Nervosa (BN) and Eating Disorder Not Otherwise Specified (EDNOS)].

Table 2 shows the descriptive statistics (means and standard errors adjusted for the covariate participants’ age) for the UPPS-P impulsivity facets according to the diagnostic subtype and the presence of the NSSI behavior. Table 3 shows the results of the ANOVA comparing this mean distribution. The interaction NSSI×group was statistically non-significant for the UPPS-P Negative Urgency and Lack of Perseverance scales, indicating that the mean differences for the NSSI factor are independent of the diagnostic subtype. For these two UPPS-P scales, participants who reported the presence of NSSI behavior obtained higher means than patients without the NSSI conduct. For the rest of the UPPS-P scales (Lack of Premeditation, Sensation Seeking and Positive Urgency), the interaction NSSI×group achieved a significant result, indicating that the mean differences based on the presence of the NSSI behavior was related to the diagnostic subtype: a) the UPPS-P Lack of Premeditation mean was higher for participants who reported the presence of NSSI behavior compared to those without NSSI only in the AN-BP subtype and b) mean scores for the UPPS-P Positive Urgency scale were higher for patients with NSSI as compared to those without NSSI into the AN-BP, BN and EDNOS subtypes. Fig 1 also illustrates how an increase in UPPS-P dimensions is related to both binge eating/purging ED subtypes and the presence of NSSI.

**Table 2:** Means (standard errors) for the UPPS-P dimensions based on the diagnostic group and the NSSI behavior adjusted for age.

	NSSI = Absent					NSSI = Present				
	HC	AN-R	AN-BP	BN	EDNOS	HC	AN-R	AN-BP	BN	EDNOS
	*N = 68*	*N = 39*	*N = 16*	*N = 67*	*N = 38*	*N = 16*	*N = 8*	*N = 10*	*N = 51*	*N = 25*
Negative urgency	25.03	25.13	32.04	35.19	29.95	28.23	28.13	37.82	36.90	34.39
	(0.80)	(1.03)	(1.61)	(0.79)	(1.06)	(1.63)	(2.28)	(2.04)	(0.90)	(1.28)
Lack premeditation	20.97	20.96	18.79	24.11	23.23	21.12	18.61	26.71	26.06	26.08
	(0.73)	(0.94)	(1.47)	(0.72)	(0.97)	(1.49)	(2.08)	(1.86)	(0.82)	(1.17)
Lack perseverance	19.34	18.47	21.25	24.00	20.39	20.39	18.92	25.34	26.63	24.81
	(0.66)	(0.86)	(1.33)	(0.66)	(0.88)	(1.35)	(1.89)	(1.69)	(0.74)	(1.06)
Sensation seeking	28.64	25.40	22.55	26.99	27.26	26.48	25.66	27.32	28.74	25.97
	(1.03)	(1.33)	(2.08)	(1.02)	(1.37)	(2.11)	(2.94)	(2.64)	(1.16)	(1.65)
Positive urgency	21.25	21.25	25.75	27.91	26.86	22.93	25.15	34.30	31.57	32.51
	(1.07)	(1.38)	(2.16)	(1.06)	(1.42)	(2.19)	(3.05)	(2.74)	(1.20)	(1.72)

NSSI = Non-Suicidal Self-Injury, HC = Healthy Controls, AN-R = Anorexia Nervosa Restrictive Subtype, AN-BP = Anorexia Nervosa Binge-eating/Purging type, BN = Bulimia Nervosa, EDNOS = Eating Disorder Not Otherwise Specified

**Table 3:** Comparison for the UPPS-P dimensions based on the NSSI behavior and the diagnostic group.

	Inter-			Factor: NSSI			
	N×G	Means for NSSI		Mean comparison for NSSI			
	*p*	Absent	Present	Mean diff.	*p*	95% CI (MD)	
Negative urgency	.547	29.5	33.1	**3.65**	**<.001**	**1.84;**	**5.41**
Lack Premeditation	.024	20.97^HC^	21.12^HC^	0.15	.927	-3.06;	3.36
		20.96^AN-R^	18.61^AN-R^	-2.35	.306	-6.86;	2.16
		18.79^AN-BP^	26.71^AN-BP^	**7.91**	**.001**	**3.27;**	**12.56**
		24.11^BN^	26.06^BN^	1.95	.075	-0.20;	4.10
		23.23^EDNOS^	26.08^EDNOS^	2.85	.061	-0.14;	5.84
Lack Perseverance	.358	20.7	23.2	**2.53**	**.001**	**1.05;**	**4.01**
Sensation Seeking	.013	28.64^HC^	26.48^HC^	-2.16	.349	-6.69;	2.37
		25.40^AN-R^	25.66^AN-R^	0.26	.937	-6.12;	6.63
		22.55^AN-BP^	27.32^AN-BP^	4.77	.154	-1.79;	11.33
		26.99^BN^	28.74^BN^	1.75	.259	-1.29;	4.78
		27.26^EDNOS^	25.97^EDNOS^	-1.29	.547	-5.52;	2.93
Positive urgency	.010	21.25^HC^	22.93^HC^	1.68	.482	-3.02;	6.39
		21.25^AN-R^	25.15^AN-R^	3.90	.247	-2.72;	10.52
		25.75^AN-BP^	34.30^AN-BP^	**8.56**	**.014**	**1.74;**	**15.37**
		27.91^BN^	31.57^BN^	**3.66**	**.023**	**0.50;**	**6.81**
		26.86^EDNOS^	32.51^EDNOS^	**5.65**	**.012**	**1.27;**	**10.04**

ANOVA controlled for age. N×G = interaction NSSI × group.

NSSI = Non-Suicidal Self Injury, HC = Healthy Controls, AN-R = Anorexia Nervosa Restrictive Subtype, AN-BP = Anorexia Nervosa Binge-eating/Purging type, BN = Bulimia Nervosa, EDNOS = Eating Disorder Not Otherwise Specified

CI = Confidence Interval MD: mean difference.

Results include Bonferroni-Simes correction for multiple significance tests.

### Predictive capacity of UPPS-P impulsivity facets on the presence of NSSI

The results of the logistic regression adjusted to the covariates age and diagnostic subtype showed that only the Lack of Perseverance achieved significant predictive capacity on the presence of NSSI (OR = 1.07, *p* =. 020) (see Table 4).

**Table 4:** Predictive capacity of the UPPS-P Impulsivity Facets on the presence of NSSI behavior: logistic regression adjusted by the covariates age and diagnostic group.

	*p*	OR	95% CI (OR)	
Age	.043	0.97	0.94;	1.00
Diagnostic group	.073	1.20	0.98;	1.46
Negative Urgency	.144	1.04	0.99;	1.09
Lack of Premeditation	.914	1.00	0.96;	1.05
Lack of Perseverance	.020	1.07	1.01;	1.13
Sensation seeking	.960	1.00	0.97;	1.04
Positive Urgency	.105	1.03	0.99;	1.07

Adjusted Nagelkerke’s-*R* ^*2*^ =. 115

OR = Odd Ratio, CI = Confidence Interval

## Discussion

In the present study, we investigated the association between NSSI and the UPPS-P model of impulsivity in a sample of eating disorder patients and healthy controls. The prevalence rates of NSSI in ED patients ranged from 17% (in AN-R) to 43% (in BN), and was situated around 19% in HC, hereby confirming international prevalence rates of NSSI in ED patients and healthy controls [3,4]. Additionally, binge-eating/purging ED patients engaged significantly more in NSSI compared to healthy controls and patients with AN, restrictive subtype.

One of the possible explanations of the higher frequency of NSSI in binge-eating/purging ED patients, is the hypothesis that binge-eating/purging ED patients are more impulsive than restrictive AN patients and healthy controls [26–28]. The findings of our study confirm this hypothesis by showing an increase in positive/negative urgency, lack of premeditation and lack of perseverance from healthy controls and AN-R patients, over AN-BP patients to Binge Eating Disorder (BED)/BN patients; hereby confirming the impulsive spectrum idea of eating disorder, which suggests an increase in impulsivity from AN-R over AN-BP to BN [26]. However, we did not find a significant difference between the different ED subtypes on Sensation Seeking which is often more associated with the enjoyment of risky behavior; than with rash action in the face of negative/positive emotions.

Similar results were found when we compared participants with and without NSSI. Participants with NSSI scored significantly higher on negative/positive urgency, lack of premeditation and lack of perseverance, confirming previous findings on the association between NSSI and impulsivity in ED patients [27,28]. The elevated negative/positive urgency scores of self-injurious participants are consistent with previous research on emotions in NSSI. Several studies have shown that self-injurious individuals are more emotionally dysregulated than individuals without NSSI, both in general community and clinical ED samples [15,19,29,30]. The Lack of Premeditation is also consistent with previous research which has consistently documented the tendency to engage in action before careful thinking and planning in individuals with NSSI [15,19,31–33]. Finally, the lack of Perseverance, indicating a tendency to remain with a task until completion and to avoid boredom might indicate a diminished ability to carry out interventions meant to stop self-injurious behavior [15].

Finally, taking into account all UPPS-P dimensions while predicting NSSI, Lack of Perseverance was the most important predictor. Problems in continuing a difficult task, such as resisting the temptation to engage in NSSI (lack of perseverance) make ED patients and healthy controls vulnerable to engagement in NSSI. Treatment of these patients and healthy controls should therefore focus on impulse regulation to resist the urge to injure (as described previously [34]). Given that lack of perseverance is the strongest predictor of NSSI, this might indicate that NSSI patients have problems with carrying out interventions to stop self-injurious behavior, considering that these interventions can evoke irritation and frustration. Therefore, guided training of impulse (and related emotion) regulation seems to be key to recovery, and this type of intervention is well described in the Dialectical Behavioral Therapy [35] and the Emotion Regulation Training [36], but also in other alternative approaches [37–40].

By training impulse (and related emotion) regulation to prevent people from engaging in NSSI, these skills can hopefully be transferred to the domain of ED symptoms, such as binging and purging, which are driven by the same impulsivity dimensions. The fact that both NSSI and BN/BED symptoms share some common impulsivity pathways can also explain the balance between these symptoms while treating them [41]. When the client is able to reduce the acts of NSSI, we often see an increase of ED symptoms; or vice versa. Therefore, the need to engage in more adaptive behaviors while facing negative and even positive experiences is a sine qua none for the treatment of both behaviors. One explanation why even positive emotions can trigger NSSI and/or BN symptoms can be that positive feelings induce a sense of discordance between the positive feelings and the often very negative self-image; to solve this discordance patients can again engage in self-destructive behaviors. Finally, we did not find a significant difference between individuals with/without NSSI with respect to Sensation Seeking, confirming the findings of previous studies [19,42].

Some limitations of the present study warrant discussion. First, the sample consists of female HC/ED participants, who represent only a subset of self-injurious individuals. Second, our study was based on self-report measures, which can increase the association between variables due to shared method variance. Finally, due to the cross-sectional design of the study, the temporal relationship of UPPS-dimensions and NSSI is unclear. For example, the performance of NSSI may dispose individuals to rate themselves as having impulsive characteristics. Therefore, future studies should include an equal ratio of male/female HC/ED individuals who are assessed by means of self-report and additional assessment techniques (e.g. performance-based measures of NSSI and impulsivity) and should investigate the association between the UPPS-P dimensions and NSSI over time to draw conclusions about the directionality of the relationship.

In sum, our study showed that binge-eating purging ED individuals showed an increased probability of engaging in NSSI which appears to be related to heightened levels of Lack of Premeditation (i.e. the inability to ignore distracting stimuli or to remain focused on particular tasks). Therefore, the treatment of both NSSI as well as eating disorders may benefit from impulse- (and related emotion) regulation interventions.
